# YOLOv7-Branch: A Jujube Leaf Branch Detection Model for Agricultural Robot

**DOI:** 10.3390/s24154856

**Published:** 2024-07-26

**Authors:** Ruijun Jing, Jijiang Xu, Jingkai Liu, Xiongwei He, Zhiguo Zhao

**Affiliations:** 1 School of Software, Shanxi Agricultural University, Taiyuan 030800, China; jrj@sxau.edu.cn (R.J.);; 2 China Nuclear Industry Huaxing Construction Co., Ltd., Nanjing 210000, China

**Keywords:** jujube leaf branch detection, efficient decoupled head, attention mechanism

## Abstract

The intelligent harvesting technology for jujube leaf branches presents a novel avenue for enhancing both the quantity and quality of jujube leaf tea, whereas the precise detection technology for jujube leaf branches emerges as a pivotal factor constraining its development. The precise identification and localization of jujube leaf branches using real-time object detection technology are crucial steps toward achieving intelligent harvesting. When integrated into real-world scenarios, issues such as the background noise introduced by tags, occlusions, and variations in jujube leaf morphology constrain the accuracy of detection and the precision of localization. To address these issues, we describe a jujube leaf branch object detection network based on YOLOv7. First, the Polarized Self-Attention module is embedded into the convolutional layer, and the Gather-Excite module is embedded into the concat layer to incorporate spatial information, thus achieving the suppression of irrelevant information such as background noise. Second, we incorporate implicit knowledge into the Efficient Decoupled Head and replace the original detection head, enhancing the network’s capability to extract deep features. Third, to address the issue of imbalanced jujube leaf samples, we employ Focal-EIoU as the bounding box loss function to expedite the regression prediction and enhance the localization accuracy of the model’s bounding boxes. Experiments show that the precision of our model is 85%, which is increased by 3.5% compared to that of YOLOv7-tiny. The mAP@0.5 value is 83.7%. Our model’s recognition rate, recall and mean average precision are superior to those of other models. Our method could provide technical support for yield estimation in the intelligent management of jujube orchards.

## 1. Introduction

Wild jujube leaves are considered to have extremely high edible value and medical value. Jujube leaves can be used to make tea, and their extracts can be used for medicinal purposes. The wild Jujube leaf tea (JLT) [1] is considered to effectively nourish the heart, soothe nerves, aid sleep, reduce blood pressure and have other effects. Researchers [2,3,4,5] utilized various methods to determine the components of the triterpenoids, saponins, flavonoids, and other compounds in the crude methanol extract of jujube leaves. The aqueous extract of wild jujube leaves exhibits similar central nervous system inhibitory effects to the wild jujube seeds used in traditional Chinese medicine. This has attracted significant attention from enterprises and other entities, leading to a gradual increase in the demand for jujube leaves. The lack of efficient and high-quality jujube leaf harvesting methods has become a key factor constraining the development of the jujube industry. The automation and intelligent harvesting of jujube leaves have become a development trend, while precise identification of jujube leaves is the key technology required for intelligent harvesting. If the system only detects the leaves, the overall efficiency of the robot is very low. Detecting the branches of the jujube leaves and processing them with picking equipment can greatly improve the efficiency of the device. We focus on the detection of jujube leaf branches in this paper.

Deep-learning technologies have made groundbreaking strides in various fields, significantly propelling the development of smart agriculture. Particularly, techniques such as semantic segmentation and object recognition have provided robust support for precision agriculture. Researchers have successively developed various detection systems, such as disease, fruit or vegetable detection [6,7]. Reference [8] leveraged multi-scale convolution to extract features such as the leaf curvature, constructing a lightweight tree species recognition model and successfully developing an app for identifying 184 tree species in the eastern United States. The advancement of real-time object detection has seen significant contributions from the YOLO (You Only Look Once) series of algorithms, where they play a pivotal role in the burgeoning field of agricultural intelligence. Researchers have successfully applied the YOLO algorithms to agricultural applications, such as YOLOv4 [9,10], YOLOv5 [11,12,13,14], YOLOv7 [15,16,17].

The aforementioned algorithms have been applied within the research domain of jujube detection and harvesting, showcasing their utility in advancing the capabilities of automated quality assessment and crop collection techniques. Zhao et al. [18] leveraged the ResNet50 architecture to devise a classifier for the quality assessment of winter jujubes. Zheng et al. [19] developed an enhanced YOLOX-Nano network for winter jujube detection, involving a diverse dataset and an attention module for feature enhancement. An enhanced YOLOv5s [20] algorithm for winter jujube detection was introduced, featuring a streamlined model, a slim-neck with Ghost-Shuffle Convolution and a Variety of View Group Shuffle Cross Stage Partial Network (VoV-GSCSP) for reduced complexity. In the referenced literature [21], a thermographic approach coupled with the DenseNet architectural framework was employed to achieve bruising detection and classification in jujube. Zheng et al. [22] designed a lightweight attention ghost high-resolution net (Ghost-HRNet) based on the architecture of semantic segmentation networks to separate jujube tree trunks and branches. A feature intersection and fusion transformer (FIT-Transformer) model [23] for visual perception was introduced, integrating diverse feature aggregation (DFA) and an attention feature fusion module (AFFM) to enhance the feature learning and accurately distinguish branches from the background.

The above discussion pertains to research on the application of deep learning in agricultural scenarios, which has achieved satisfactory results. Inspired by these studies and the rapid development of picking robots, this paper focuses on the jujube branch detection and positioning algorithm for jujube branch-picking robots. In practice, it was found that the labeling of jujube leaf branches introduced background noise (this part is analyzed and elaborated in detail in Section 2). Background noise, occlusion, and morphological differences pose significant challenges to the detection of jujube branches. To address these issues and enhance the algorithm’s accuracy, the main works are undertaken, which are summarized as follows:

(1) The Polarized Self-Attention module is embedded into the convolutional layer, and the Gather-Excite (GE) follow-up is embedded into the concatenation layer, so as to enhance the network’s feature extraction for jujube leaves and to improve the detection accuracy.
(2) The Efficient Decoupled Head based on YOLOR’s implicit knowledge is proposed, and we use it to replaces the detection head from the original YOLOv7-tiny model, so as to extract deep information from the network.
(3) The focal and efficient intersection over union (Focal-EIoU) loss calculation replaces the complete intersection over union (CIoU) calculation, making the model increasingly accurate when the jujube leaf branches are detected.

While ensuring detection accuracy, the parameters of the network model and the floating-point operation (FLOPS) are greatly reduced, and the calculation and size of the model are reduced.

## 2. Data Acquisition and Preprocessing

### 2.1. Data Acquisition

Under various weather conditions, we collected 2000 images of jujube leaf branches. They were captured using four smartphones from March to July 2023 at the National Jujube Germplasm Resources Institute of the Shanxi Agricultural University Fruit Tree Research Institute, Taigu County, Shanxi Province, China. The orchard is predominantly devoted to the cultivation of Taigu Bottle Jujube. The related parameters of the smartphones’ cameras are shown in Table 1.

### 2.2. Data Comparative Analysis

**Figure 1:**
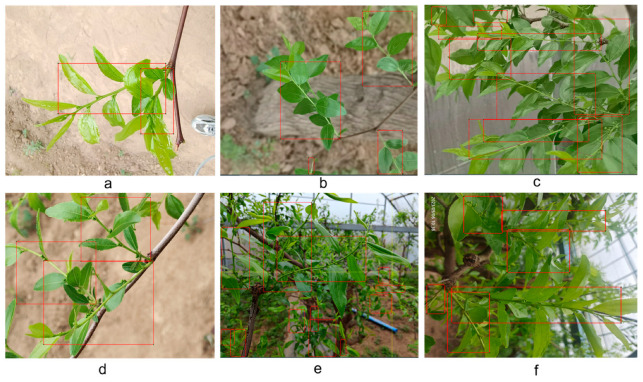
Jujube leaf images and labels. (**a**) example 1, (**b**) example 2, (**c**) example 3, (**d**) example 4, (**e**) example 5, (**f**) example 6.

Figure 1 presents partial images of jujube leaf branches, along with their labeled versions. The data were collected under various weather conditions, during the stage of jujube leaf bud development. Comparing Figure 1a,b,e, there are certain morphological differences in the jujube leaf branches. Comparing Figure 1c,f, the shooting angles differ, leading to varied representations of their form.

In Figure 1a,b,d, the red boxes represent the labels. In Figure 1d, the area of the background within the largest labeled frame is larger than the portion occupied by the jujube leaf branch. During the heat map analysis of the model, it was found that the background parts of the images were focused (as shown in Figure 6). In Figure 1c,d,f, it can be observed that the jujube leaves are occluded, and in Figure 1e,f, the jujube leaves in the distance are blurry. This makes the labeling process challenging and increases the difficulty of feature extraction.

### 2.3. Data Enhancement

To further mitigate the detection challenges and address the impact of the dataset quality on the performance and generalization of the machine-learning models, various data augmentation techniques were employed.

Rotational and mirroring transformations were applied to the original images, resulting in data augmentation. This process expanded the original dataset of 2000 images to a total of 16,000 images. We utilized 11,200 images as training data, 3200 images for validation, and 1600 images for testing.

## 3. Methods

### 3.1. YOLOv7 Algorithm

In 2022, Chien-Yao Wang [24] introduced the YOLOv7 algorithm, a state-of-the-art object detection method. The algorithm is optimized through model architecture reparameterization and dynamic label assignment strategies, achieving a balanced trade-off between model accuracy and inference performance. Various versions, such as YOLOv7 and YOLOv7-tiny, have been released, which are better equipped to support mobile GPU edge devices and high-computing-power GPU devices.

The number, depth, and width of the feature layers vary across different versions of the YOLOv7 network architectures. Considering the balance between the computational load and detection accuracy, this paper adopts YOLOv7-tiny as the base model. YOLOv7-tiny significantly reduces the parameters and FLOPs, while ensuring the high detection accuracy of the model. 

YOLOv7-tiny employs a design based on Feature Pyramid Networks (FPNs), merging feature maps of different scales, enabling the model to better adapt to objects of varying sizes and shapes, thereby enhancing the detection accuracy. YOLOv7-tiny uses an anchor-free approach, eliminating the need to predetermine the anchor boxes, making the algorithm more streamlined and efficient. YOLOv7-tiny utilizes Mish as the activation function, which more effectively addresses the issues of gradient vanishing and exploding, resulting in more stable and efficient model training. 

### 3.2. Improvement of the YOLOv7-Tiny Model

**Figure 2:**
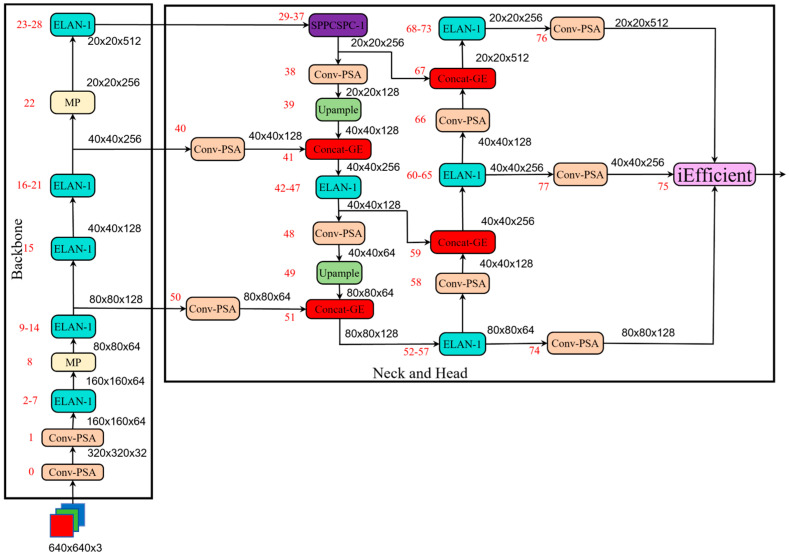
The improved YOLOv7-tiny algorithm architecture.

Although the YOLOv7-tiny algorithm has achieved notable results in object detection, there are deficiencies in recognizing jujube leaves, mainly due to issues like the occlusion characteristic of jujube leaves. In light of this, this paper proposes the integration of the Polarized Self-Attention (PSA) mechanism into the convolutional module; the incorporation of spatial attention mechanisms into the concatenation module to suppress irrelevant information and extract relevant information; the use of an Efficient Decoupled Head to extract key implicit knowledge hidden deep within the neural network; and the adoption of Focal-EIoU as the bounding box loss function to accelerate the regression prediction. This paper enhances the base algorithm by incorporating attention mechanisms, among other methods, with the optimized model presented in Figure 2.

The network comprised a backbone network, a neck network, and a head network. The backbone network comprised the Conv + BN + Silu + PSA (Conv-PSA) module, efficient long-range attention network (ELAN) module, and MaxPool (MP) module. The neck network adopted the PANet network structure, which was a top-down and bottom-up bidirectional fusion backbone network. It comprised the Conv-PSA module, efficient aggregation network module and Concat + Gather-Excite (Concat-GE) module. The incorporation of spatial attention mechanisms into the concatenation module served to suppress irrelevant information and extract relevant information. It realized the multiscale fusion of the network by aggregating the characteristics between different backbone network layers and detection layers. The head network employs an Efficient Decoupled Head to extract key implicit knowledge hidden deep within the neural network. It convolves outputs from three different scale feature maps, producing three feature maps of sizes 20 × 20 × 512, 40 × 40 × 256, and 80 × 80 × 128. Larger feature maps are used for detecting smaller objects, while smaller feature maps are used for detecting larger objects, thereby enabling object detection at different scales and further improving the model accuracy.

#### 3.2.1. Attention Module

The attention mechanism is inspired by biomimetic vision, selectively focusing on the primary information of events. It intensifies the concentration on regions of interest, effectively suppressing irrelevant information, thereby enhancing the efficiency and accuracy of the algorithm. In this paper, we propose the integration of the Polarized Self-Attention mechanism [25] into the convolutional module and the incorporation of the Gather-Excite algorithm into the concatenation layer, enhancing YOLOv7-tiny’s capability to capture feature information.

1. Polarized Self-Attention

The phenomenon of cross-overlapping among jujube leaves results in a lower object recognition rate. The PSA algorithm is designed specifically for fine-grained pixel-level tasks. With a low computational overhead, it models the long-distance dependencies of high-resolution input/output, thereby estimating highly nonlinear semantic information. High-resolution input is a solution to the object overlap issue and an effective approach to enhance the model accuracy. In this paper, we choose the PSA algorithm to optimize the structure of YOLOv7.

**Figure 3:**
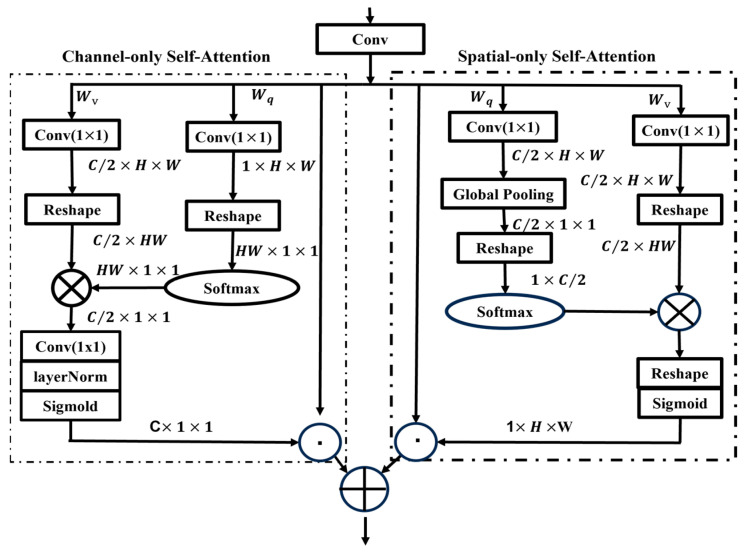
The structure of the Polarized Self-Attention mechanism.

In CNNs, the attention mechanism can capture the dependencies over lengths, but this method is relatively complex and sensitive to noise. Given the intricate conditions of agricultural fields, the reliability, stability, and accuracy of this method in solving practical problems are challenging to guarantee. In this paper, we integrate the PSA mechanism into the convolutional module to extract high-resolution semantic information concerning jujube leaves. The framework of the optimized algorithm is depicted in Figure 3.

The integration of the PSA algorithm ensures both the channel and spatial dimensions, with the channel dimension being C/2 and the spatial dimension [H W], minimizing the loss of semantic information due to dimension reduction.

2. Gather-Excite

**Figure 4:**
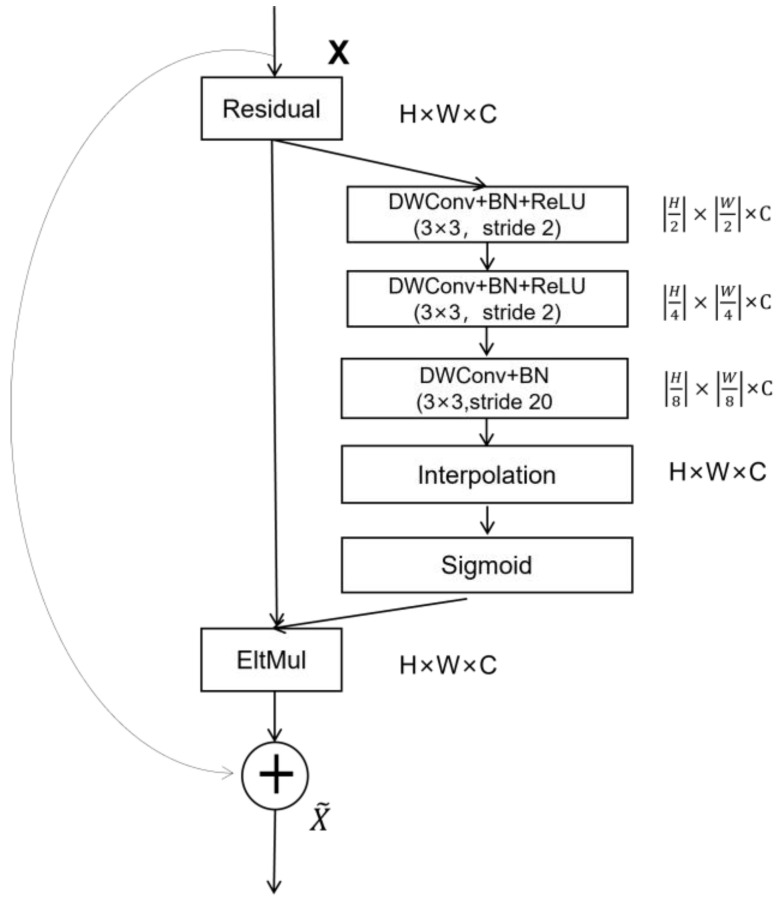
Structure of the Gather-Excite mechanism.

Gather-Excite [26] is a simple, lightweight spatial attention mechanism that utilizes contextual information. Gather uses strided depth-wise convolution as the gather operator, which applies spatial filters to the independent channels of the input, effectively aggregating the feature responses over a large spatial extent. The Excite operator then resizes the aggregates, applies a sigmoid and multiplies the result with the input. The aggregated information can be redistributed to local features. The framework of the algorithm is depicted in Figure 4.

Here, interp(·) denotes resizing to the original input size via nearest neighbor interpolation. $x=\left\{x^{c}:c\in \left\{1,\ldots ,C\right\}\right\}$ denotes a collection of feature maps produced by the network. $\xi_{G}{\left(x\right)}_{u}^{c}=\xi_{G}(x⨀1_{l_{\left(u,e\right)}}^{c})$, where ⨀ is the Hadamard product. $l\left(u,e\right)=\left\{eu+\delta :\delta \in [-[(2e-1)/2],{\left[(2e-1)/2\right]}^{2}]\right\}$ is the selection operator. e represents the extent ratio of the selection. In terms of the number of parameters added and the computational complexity, these operators are inexpensive and can be directly integrated into existing architectures to enhance their performance.

#### 3.2.2. Efficient Decoupled Head

In YOLOvR, implicit knowledge learning is employed to learn deep feature information from data. The implicit knowledge learning in YOLOvR [27] is divided into two stages: pre-training and fine-tuning. During the pre-training stage, the model is trained with a large amount of unlabeled data and uses a contrastive loss algorithm to extract features. In the fine-tuning stage, the model uses labeled data to fine-tune the features obtained from the implicit knowledge learning. The YOLOv7-tiny architecture employs a detection head with implicit knowledge learning during its training phase.

In the field of object detection, the first-stage and second-stage detectors for classification and localization tasks often utilize structured heads. The detection head of YOLOv5 is a coupled head with parameters shared between the classification and localization branches. On the other hand, YOLOX [28] enhances the structured head by adding two 3 × 3 convolutional layers to each branch to boost the performance.

YOLOv6 [29] adopts a hybrid channel strategy to build the Efficient Decoupled Head. These modifications further reduce the computation costs to achieve a lower inference latency. EfficientDet, proposed by Google, is an efficient object detection algorithm that incorporates the ideas of implicit knowledge learning and decoupled heads. Unlike YOLOvR, EfficientDet introduces the bi-directional feature pyramid network (Bi-FPN) to optimize the feature pyramid network and employs different decoupled heads for different levels of object detection, which improves the accuracy of modeling the hierarchical relationships of the targets.

**Figure 5:**
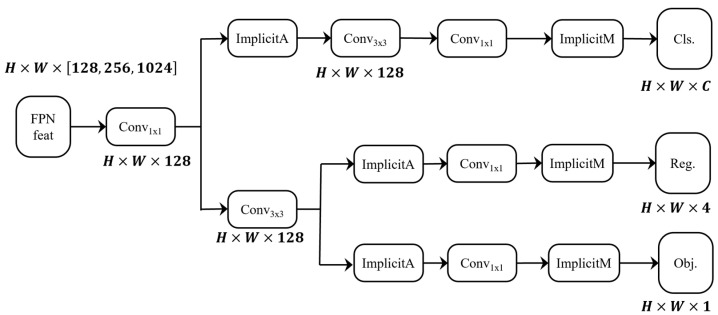
The Efficient Decoupled Head.

Based on the aforementioned research, this paper draws inspiration from the implicit knowledge learning concept of the YOLOv7-tiny model and proposes an Efficient Decoupled Head with implicit knowledge learning. The algorithm’s structure is illustrated in Figure 5.

**Figure 6:**
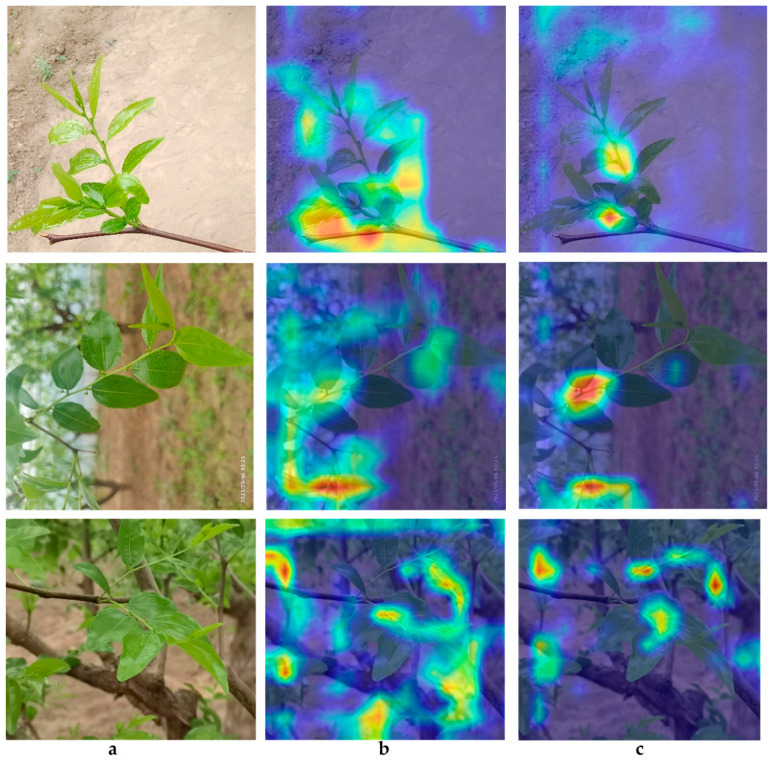
(**a**) Original image, (**b**) heatmap generated by YOLOv7-tiny, and (**c**) heatmap generated by our algorithm.

It can extract the implicit information of jujube leaf branch features. Figure 6 shows the heat maps before and after the modification of the detection head. Figure 6a is the original image, Figure 6b is the heat map generated by the original algorithm, and Figure 6c is the heat map after replacing the detection head. It can be observed that the improved heat map shows better effectiveness in extracting the features of the jujube leaf branch. Before the improvement, the features around the jujube leaf branch were also extracted, which had a significant impact on the accuracy of the identification. The improved heat map shows that the features are more concentrated, which can effectively identify jujube leaf branches.

#### 3.2.3. Loss Function

For object detection tasks, the loss function is a crucial component, as it directly determines the performance and accuracy of the model. The higher the value of the loss function, the greater the error between the predicted frame and the actual frame.

To render the predicted frame closer to the actual frame and improve the detection effectiveness, we replaced the original CIoU loss function with Focal-EIoU as the loss function for jujube leaf branch identification.

**Figure 7:**
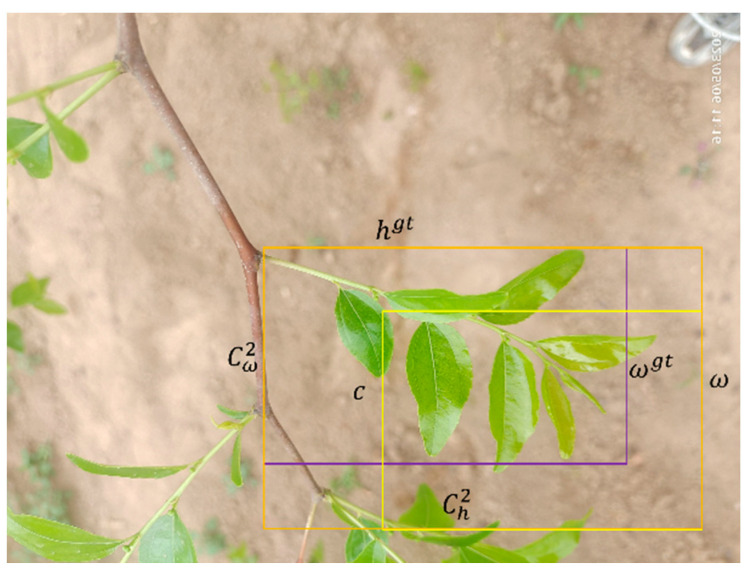
The EIoU loss function graph.

Efficient intersection over union (EIoU) is a loss function that directly penalizes the predictions of width *ω* and height h, effectively addressing the issue of edge length magnification encountered in distance intersection over union (DIoU), as showed in Figure 7. It is defined as follows:

(1) $$L_{EIoU}=L_{IoU}+L_{dis}+L_{asp}=1-IoU+\frac{\rho^{2}\left(b,b^{gt}\right)}{c^{2}}+\frac{\rho^{2}\left(\omega ,\omega^{gt}\right)}{C_{\omega }^{2}}+\frac{\rho^{2}\left(h,h^{gt}\right)}{C_{h}^{2}}$$

Here, $C_{\omega }$ and $C_{h}$, respectively, represent the width and height of the enclosing rectangle. The EIoU divides the loss function into components: $L_{EIoU}$ means the loss of overlapping between the predicted frame and the true frame, $L_{dis}$ means the loss of the center distance between the predicted frame and the true frame, and $L_{asp}$ means the loss of the width and the height between the predicted frame and the true frame.

Upon analyzing the heat map, it was found that the anchor boxes contained background and overlapping information, leading to lower recognition accuracy. Integrating the Gather-Excite (GE) attention module effectively mitigated the influence of the background but did not eliminate it. We adopt Focal-EIoU [30] to replace the loss function in YOLOv7, as shown in the following formula:

(2) $$L_{Focal\text{-}EIoU}={IoU}^{\gamma }L_{EIoU}$$

Here, the hyperparameter *γ* is used to control the curvature of the curve, addressing the issue of the imbalance between difficult and easy samples.

Focal-EIoU approaches the problem from the perspective of classifying samples by difficulty, focusing the loss on hard-to-distinguish samples and addressing the issue of low classification accuracy for categories with fewer samples. Notably, hard-to-distinguish samples are not limited to categories with fewer instances. Therefore, the focal loss not only solves the problem of sample imbalance but also contributes to the overall performance improvement of the model.

## 4. Experiments and Results

### 4.1. Evaluation Index and Platform

#### 4.1.1. Evaluation Index

In this study, the average precision (AP), precision (P), and recall (R) are used to evaluate the model’s performance in detecting jujube leaf branches. These metrics are utilized as performance indicators for comparison with other models.

True positives are instances that correspond to the actual targets, while false positives are instances that do not correspond to the actual targets. The AP is the primary metric for evaluating the model’s detection performance. The P represents the ratio of true positives predicted by the model to the total predicted positives, reflecting the model’s false positive rate. The R represents the ratio of true positives predicted by the model to the actual positives in the image, reflecting the model’s false negative rate.

The formulas for the AP, P, and R are as follows:

(3) $$AP=\frac{\sum_{i=1}^{n}PR}{k};P=\frac{TP}{TP+FP};R=\frac{TP}{TP+FN}$$

Here,

TP (true positives) refers to the number of actual positive cases correctly predicted as positive.

FP (false positives) refers to the number of actual negative cases incorrectly predicted as positive.

FN (false negatives) refers to the number of actual positive cases incorrectly predicted as negative.

#### 4.1.2. Experimental Platform

All the experiments in this study were conducted using the PyTorch deep-learning framework and were programmed in Python 3.8.0. The main specification of the computer used in the experiments was a 13th Gen Intel(R) Core (TM) i9-13900K CPU (Santa Clara, CA, USA). The running memory was 32 GB RAM, the operating system was Ubuntu 20.04, and the GPU was NVIDIA GeForce RTX 4070 Ti 12G (Santa Clara, CA, USA). The specific settings of the parameters were as follows: the learning rate was 0.001, the optimizer weight decay factor was 0.0005, the total number of epochs trained was 300, the batch size was 8, the number of works was 4, and the input image size of the model was 640 × 640 pixels.

### 4.2. Comparison of Different Network

#### 4.2.1. Ablation Experiments

The effectiveness of each component is demonstrated through ablation studies, with the results presented in Table 2. From the experimental results, incorporating the PSA mechanism into the convolutional part of the model resulted in a 0.4% increase in precision (P) and a 0.2% increase in the mAP@0.5:0.95.

By integrating the GE attention module into the concatenation layer, the model’s recall (R) value increased by 0.7%, and the mAP@0.5:0.95 increased by 0.3%. The inclusion of attention mechanisms in the model enhances its feature extraction capability.

By replacing the detection head with an Efficient Decoupled Head capable of implicit knowledge learning, improvements were observed in the model’s precision (P), mAP@0.5:0.95, and mAP@0.5. Specifically, the model’s P increased by 2.9%, its mAP@0.5 by 0.8%, and its mAP@0.5:0.95 by 0.9%. The overlapping of jujube leaves is a key factor affecting their recognition. Changing the detection head effectively enhanced the extraction of implicit feature information from the jujube leaves, significantly improving the model’s recognition capability.

When modifying the loss function, the model achieved a 3.5% increase in precision, a 0.8% increase in mAP@0.5, and a 1.2% increase in mAP@0.5:0.95. This improvement was primarily due to the separation of the loss components into the differences in the predicted width and height and the minimum external frame width and height, which accelerated the convergence speed and enhanced the regression accuracy.

The introduction of focal loss optimized the sample imbalance in the task of bounding box regression. It reduced the number of anchor frames with less overlap with the target frame in the BBox regression, focusing the regression process on high-quality anchor frames.

The ablation studies indicate significant improvements in four metrics of the model before and after the modifications. Through the attention module, loss function, and improved detection head, the YOLOv7 model’s precision (P) for jujube leaf detection increased from the original 81.5% to 85%, achieving better results.

#### 4.2.2. Comparison of Different Networks

To demonstrate the advantages of our model, we compared its performance with other common object detection algorithm models, including the classic Fast-RCNN and other YOLOv series models. The comparative experimental results are presented in Table 3.

From the table, it can be observed that, overall, our model is very effective in terms of the accuracy and recall for detecting jujube leaves, with an mAP of 83.7%. Our model showed a 35% higher accuracy compared to YOLOv7 and YOLOv7-tiny, but it experienced a 0.3% decrease in recall compared to YOLOv7. This indicates that the improved network reduced the occurrence of missed detections in the dataset.

Considering the dataset of jujube leaves, the overlapping of the leaves is a limiting factor in their detection. In comparison, the improved network, by incorporating attention mechanisms and replacing the detection head, enhanced the recognition of jujube branch features. Other YOLO series models, such as YOLOv5 and YOLOv8, also support this finding. The YOLOv8 and YOLOv7-tiny algorithms have fewer floating-point operations, but their recognition rates are 3% lower than our model, and all the other metrics are also lower than our model. Due to the overlapping of jujube leaves, the overall recognition rate of the jujube leaf identification models is relatively low.

**Figure 8:**
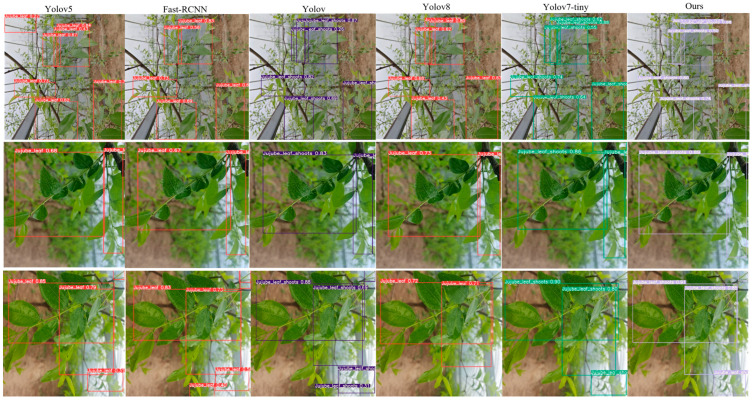
Comparison of target detection.

Figure 8 displays the detection results of the algorithms. The precision data in the image indicates that our proposed algorithm has a significant advantage. In the bottom row, both Fast-RCNN and YOLOv7 exhibit issues in recognizing remote and blurred jujube tree branches. In the top row, there are still misrecognition problems in the upper right corner of YOLOv5’s recognition results. Regarding the size of the bounding boxes for object detection, our proposed algorithm more accurately conforms to the shape of the jujube tree branches. From the recognition results, taking the bottom row as an example, our recognition results are 91% and 85%. The recognition rates of the other algorithms are as follows: YOLOv5: 85% and 79%, Fast-RCNN: 83% and 73%, YOLOv7: 88% and 69%, YOLOv8: 72% and 71%, YOLOv7-tiny: 90% and 80%. Although our model is relatively large, its recognition rate, recall, and mAP are superior to the other models, meeting the needs of engineering applications.

#### 4.2.3. Experimental Comparison before and after Model Improvement

The confidence threshold for all the experiments is set to 0.5 for comparison. The experimental results are presented in Table 4. The improved YOLOv7-tiny model, compared to the original YOLOv7-tiny model, shows an increase of 11.3% in precision (P), an increase of 28.1% in recall (R), and an improvement of 42.5% in mAP@0.5. Relative to the original YOLOv7-tiny model trained with the enhanced dataset, the improved YOLOv7-tiny model shows an increase of 3.5% in P, an increase of 0.3% in R, and an improvement of 1.2% in mAP@0.5.

**Figure 9:**
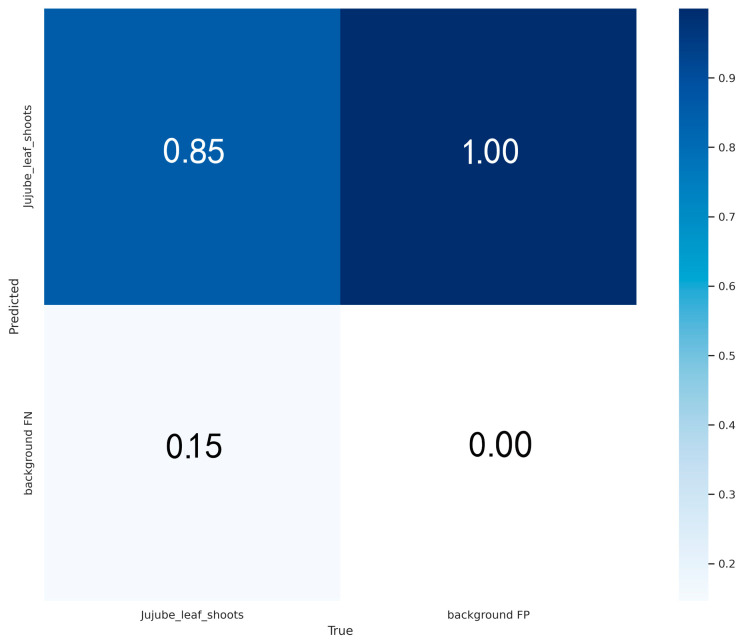
The confusion matrix of our algorithm.

Figure 9 presents the confusion matrix of our algorithm’s object detection performance. The matrix reveals that our model misclassified the target jujube leaf shoots as background at a rate of 15%. Upon comparison with the labeled images in Figure 1 and the heatmap in Figure 6, it is evident that the data-labeling process has inadvertently incorporated background noise characteristics. These noise features, which are distinct from the target jujube leaf shoots, have been extracted by the algorithm and incorrectly labeled as the target class.

**Figure 10:**
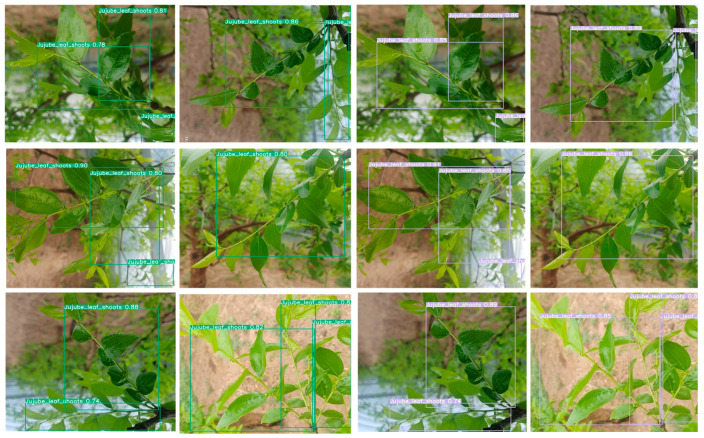
The left two columns show the detection results of YOLOv7-tiny, while the right two columns show the detection results of our algorithm.

Some experiments were conducted on the industrial computer of a robot with an i7 CPU, achieving a detection time of 34.5 ms, which meets the real-time requirements of the picking site. The detection performance of the improved model for small targets and partially occluded targets has also been enhanced. The experimental results for six randomly selected images of young jujube leaf branches demonstrate this improvement. The improved model shows a notable improvement in reducing the missed detections of young jujube leaf branches, with specific results illustrated in Figure 10.

#### 4.2.4. Application of Our Method

Based on the proposed method, we developed a supervisory application for jujube leaf detection. It can complete the detection and location of jujube leaf branches. This software can acquire data from the camera, enabling real-time detection and localization of jujube leaves.

**Figure 11:**
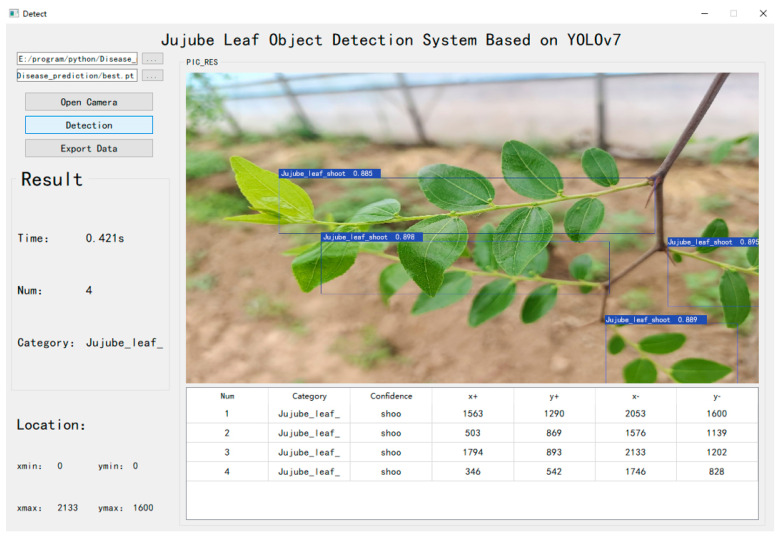
Application of jujube leaf detection.

From Figure 11, it is evident that the software displays detection information for each frame, which includes the detection time, label values, and quantity information.

## 5. Conclusions

To meet the requirements of jujube leaf branch detection and localization for jujube leaf-picking robots, we explored a jujube leaf branch detection and localization system based on object recognition. However, challenges such as the background noise from tags, occlusions, and variability in leaf morphology impede the accuracy and precision of these systems.

To address these problems, we introduced a novel jujube leaf branch detection network based on YOLOv7-tiny, incorporating several advanced techniques to refine the detection accuracy.

We adopted the method of adding multiple attention mechanisms to enhance the model’s feature extraction capability, introducing Polarized Self-Attention in the convolutional layers and incorporating Gather-Excite (GE) in the concatenation layers. This enhances the network’s spatial information-processing capabilities, further aiding in the accurate localization of jujube leaf branches. We integrated implicit knowledge learning on top of the Efficient Decoupled Head, replacing the original detection head to extract implicit knowledge hidden deep within the network. The Focal-EIoU loss function was adopted to replace the original loss function, enhancing the model’s boundary localization accuracy. The experimental results demonstrated a notable improvement in the precision (85%) and mAP@0.5 (83.7%), surpassing the performance of the YOLOv7-tiny model by 3.5%. These enhancements render the model not only more efficient but also more effective than other existing models in terms of the recognition rate, recall, and mean average precision. 

The jujube leaf branch detection model serves as the core component for the intelligent operation of jujube leaf-picking robots. The algorithm provides boundary information (location and size) and confidence levels for detection. By applying conditional constraints to the detection information and other methods, we facilitate the planning of the robotic arm’s motion path, thereby enabling the execution of jujube leaf-picking tasks.

## Figures and Tables

**Table 1:** Four smartphones’ camera pixels and the number of images.

	Camera Pixel	Number of Images
iQOO9 pro (Vivo, Dongguan, China)	50-megapixel	621
Huawei mate40 (Shenzhen, China)	50-megapixel	516
Huawei nova9 (Shenzhen, China)	50-megapixel	423
Redmi K60 Pro (Xiaomi, Beijing, China)	54-megapixel	440

**Table 2:** Results of the ablation experiments.

Model	Yolov7	PSA	GE	Efficient	Focal-IOU	P	R	mAP@.5	mAP@.5:95
Model1	✓					81.5	76.4	82.5	38
Model2	✓	✓				81.9	76.8	82.5	38.2
Model3	✓	✓	✓			80.8	77.3	82.7	38.2
Model4	✓	✓	✓	✓		84.4	74.4	83.3	38.9
Model5	✓	✓	✓	✓	✓	85	76.7	83.7	39.2

**Table 3:** Comparison of target detection.

Model	P	R	mAP@.5	mAP@.5:0.95	FLOPs
YOLOv5	79.2	75.7	80.9	37.6	16.5
Fast-RCNN	75.3	72.1	73.4	35.6	203.2
YOLOv8	81.3	73.5	80.6	39.4	8.6
YOLOv7	81.5	77	82.9	38.7	103.2
YOLOv7-tiny	81.5	76.4	82.5	38	13
Ours	85	76.7	83.7	39.2	31.6

**Table 4:** Comparison before and after model improvement.

	P	R	mAP@0.5
YOLOv7-tiny-original	73.7	48.6	41.2
YOLOv7-tiny-enhanced	81.5	76.4	82.5
Ours	85	76.7	83.7

## Data Availability

Given that the data used in this study were self-collected, the dataset is being further improved.
